# Promoting Active Citizenship in Mathematics and Science Teaching

**DOI:** 10.1007/s10763-021-10182-1

**Published:** 2021-06-19

**Authors:** Katja Maass, Stefan Sorge, Marta Romero-Ariza, Alice Hesse, Oliver Straser

**Affiliations:** 1 International Centre for STEM Education at University of Education Freiburg, Kunzenweg 21, 79117 Freiburg, Germany; 2 Department of Physics Education, IPN – Leibniz-Institute for Science and Mathematics Education, Olshausenstr. 62, 24118 Kiel, Germany; 3 University of Jaén, Campus Las Lagunillas S/N, 23071 Jaén, Spain

**Keywords:** Mathematical modeling, Inquiry-based learning, Socio-scientific issues, Teachers’ professional development, Design research

## Abstract

The world is facing severe global challenges such as climate change, food security, rising migration, social justice, or the current corona crisis. In these times, citizenship education seems more important than ever. How can this citizenship education relate to mathematics and science learning? The research project MaSDiV (Supporting mathematics and science teachers in addressing diversity and promoting fundamental values) connected mathematics and science with citizenship education by modeling real-life problems relevant to society. In this paper, we present the foundational design features of the PD course as well the results from the accompanying evaluation of this PD course, which was implemented by partners in six countries to support teachers in connecting mathematics and science education with citizenship education. More specifically, we investigate how participating teachers experienced the PD program; how their self-efficacy beliefs, learning-related beliefs, as well as teaching practices change; and which factors contributed to that change. In order to investigate the outcome of the PD program, we surveyed *N* = 311 mathematics and science teachers’ pre- and post-participation of the PD in six different European countries. Among others, our results show that in general, most participating teachers reported a high overall satisfaction with the course across all six participating countries. They also indicate that teachers’ self-efficacy beliefs about using relevant contexts, their learning-related beliefs about the benefits of using contexts, as well as their own teaching practice changed significantly after participating in the MaSDiV PD course.

**Supplementary Information:**

The online version contains supplementary material available at 10.1007/s10763-021-10182-1.

## Introduction

The world is facing severe global challenges such as climate change, food security, rising migration, social justice, or the current corona crisis. Such challenges affect fundamental values of freedom, democracy, as well as human rights, and therefore, we need a society capable of finding adequate solutions for global challenges whilst respecting fundamental values. In this context, the European Commission emphasizes the necessity of ensuring that young people acquire social, civic, and intercultural competences (European Commission/EACEA/Eurydice, 2016). These core competences can be established by promoting active citizenship as well as enhancing critical thinking, media literacy, and key competences in STEM subjects (European Commission, 2019; European Commission/EACEA/Eurydice, 2016).

However, in relation to these requirements, we face several challenges: First, in Europe, 17% of 15-year-olds underachieve in science and 22% in mathematics (Official Journal of the European Union, 2015). Second, in particular in relation to mathematics, we are faced with the so-called relevance paradox (Niss, 1992): mathematics is both pervasive and invisible. The increase in technology enhances the relevance/role of mathematics, but at the same time, mathematics is mainly hidden in tools which function as black boxes for its users and we therefore do not see its relevance (Niss, 1992). Third, science (including mathematics) education has focused on the “learning of science” (Hazelkorn et al., 2015), on pure science detached from societal implications. This focus can be contrasted with learning “of and about science” (Osborne & Dillon, 2008). Learning about science includes the social, cultural, and ethical dimensions of science (e.g., decision-making in genetic engineering), which are often neglected in classrooms (Maass et al., 2019a). Learning of and about science also fosters young people’s understanding of nature, applications, and implications of science (Hazelkorn et al., 2015). Consequently, by learning of and about science, students learn principles vital in democratic and pluralistic European societies. In this sense, science and mathematics education can also become part of citizenship education.

The idea of connecting science and mathematics education with citizenship education is not new. In mathematics education, scholars already discussed the notion of mathematical modeling and bringing extra-mathematical contexts into lessons in the twentieth century (for an overview see, e.g. Burkhardt, 2018). In relation to science education, research proposes the engagement of socio-scientific issues (SSI) as one promising path to developing citizenship competences. SSI “are controversial, socially relevant, real-world problems that are informed by science and often include an ethical component” (Sadler et al., 2007). When implementing both modeling and SSI in lessons, we use inquiry-based learning (IBL), a student-centered learning approach, in which students are actively involved in inquiry-related processes.

Connecting mathematics and science education with citizenship education trough modeling, SSI as well as IBL, can be an answer to all three challenges mentioned above. It results in students from diverse backgrounds learning about science and mathematics; it shows the relevance of mathematics and science for society and students, which in turn can raise students’ performance; and it shows students the nature of science.

The research project MaSDiV (Supporting mathematics and science teachers in addressing diversity and promoting fundamental values, 2017–2020) followed the approach of connecting mathematics and science education with citizenship education. Within the project, partners from six countries designed a professional development (PD) course to support teachers in connecting mathematics and science education with citizenship education by using contexts relevant to society.

In this paper, we focus on presenting the basic principles that guided the development of the PD course materials (i.e. modeling, SSI, and IBL) and answering the following three research questions to evaluate the outcome of the project MaSDiV:

- How do science and mathematics teachers perceive a professional development program that focuses on connecting science and mathematics education with citizenship education?
- How do science and mathematics teachers’ self-efficacy, learning-related beliefs, and practices for using relevant contexts change over the course of a professional development program?
- Which factors impact the development of teachers’ self-efficacy, learning-related beliefs and practices for using contexts?

In the following, we first turn to the theoretical background of the study. We have a look at mathematical modeling and socio-scientific issues as a basis for connecting mathematics and science with citizenship education and extend that perspective to inquiry-based teaching methods for implementation in lessons. We will also have a look at the theoretical background in relation to professional development. Then, we turn to the PD concept we used in MaSDiV. Afterwards, we describe the design of the study and finally, the results in relation to our three research questions are outlined.

## Theoretical Background

### Mathematical Modeling

**Fig. 1:**
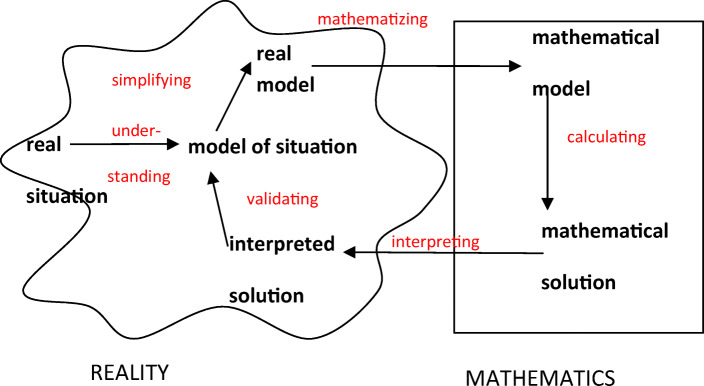
An idealized scheme of the modeling process (according to Blum and Leiß, 2007)

There are many different definitions of mathematical modeling (Kaiser & Sriraman, 2006). In this paper, we regard mathematical modeling as solving an extra-mathematical problem from the real world by carrying out a modeling process (Niss et al., 2007) and taking the modeling process as outlined in Fig. 1 as a basis.

Modeling tasks need to have an authentic, extra-mathematical character (cf. Kaiser et al., 2011). Palm (2007) defines authenticity of problems as “being true” in relation to whether the problem, taken from a situation in the real world, has occurred or might happen.

Mathematical modeling can motivate students to engage more deeply in mathematics and develop a realistic perspective on mathematics (Kaiser, 1995), foster mathematical and scientific literacy (Steen, 2001), and foster the development of students’ civic competences (Artigue & Blomhøj, 2013).In addition, modeling activities have a positive impact on students’ competence in applying mathematics to complex situations (see, e.g. Maass, 2007), on mathematical competences (e.g. English & Watson, 2018) as well as on transversal competences (e.g. Ärlebäck & Doerr, 2018).

The development of civic and transversal competences requires the selection of appropriate situations and problems. Such problems should include decision making and ethical, moral, social or cultural aspects. Unfortunately, these aspects are often not explicitly mentioned in discussions about mathematical modeling. Discussions about decision-making and controversy are allocated to socio-scientific issues (Sadler, 2011).

### Socio-scientific Issues

Socio-scientific issues (SSIs) engage students in dialogs, discussions, and debates based on science. They have a controversial nature and students need to form an opinion and make a decision on this issue. This decision might include taking moral, ethical, or social aspects into account (Zeidler & Nichols, 2009). Naturally, such issues serve primarily the purpose of educating for scientific citizenship (Owen et al., 2012). A typical example of an SSI is the question whether a shutdown to overcome COVID-19 is/was reasonable or not. Research has shown that SSIs can be used as contexts for learning scientific content (Applebaum et al., 2006; Walker, 2003; Zohar & Nemet, 2002), for understanding the nature of science (learning “about science”; see Osborne and Dillon, 2008), and for citizenship education (Herman et al., 2018; Radakovic, 2015; Sadler et al., 2007).

Theoretical lenses such as modeling and SSI in mathematics or science education can provide pathways of choosing relevant problems but they do not refer to specific pedagogies that should be implemented in science and mathematics classrooms. One approach that has shown to be appropriate for dealing with relevant problems is inquiry-based learning (IBL) (Knippels & van Dam, 2017). Consequently, combining modeling, SSI, and IBL approaches seems to have the potential to promote active citizenship in mathematics and science education.

### Inquiry-Based Learning (IBL)

IBL is a student-centered learning approach, in which students are involved in inquiry-related processes such as observing phenomena and creating their own questions, selecting mathematical or scientific approaches, creating representations to clarify relationships, seeking explanations, interpreting and evaluating solutions, as well as communicating those solutions (Dorier & Maass, 2014). The teacher makes constructive use of students’ knowledge, challenges them with probing questions, manages small group and whole class discussions, helps students to make connections between their ideas, and encourages them to consider alternative viewpoints (Swan, 2007).

This active stance that students take, as promoted in IBL, serves the purpose of citizenship education. It supports the development of critical thinking and decision-making skills, learning to consider ethical, social, and cultural aspects, and learning to deal with controversy (Geiger et al., 2015; Zeidler & Nichols, 2009). The connection between IBL and education serving democracy was also pursued in the EU-project PARRISE (Knippels & van Dam, 2017). For the connection between IBL and SSI, PARRISE identified a particular need for professional development (Knippels & van Dam, 2017). Teachers need additional learning opportunities to choose relevant, up-to-date contexts and embed them with IBL pedagogy in their classrooms (Romero-Ariza et al., 2018).

### Professional Development of Teachers

When we talk about professional development (PD) of teachers, we relate it to growth in teachers’ professional knowledge (Shulman, 1986). Additionally, PD should also attend to teachers’ classroom practice (Clarke & Hollingsworth, 2002), as well as their motivation, self-regulation, and beliefs (Baumert and Kunter, 2013). Teachers’ beliefs comprise of implicit and explicit notions about teaching and learning (Pajares, 1992). Teachers’ motivation and self-regulation are strongly connected to teachers’ self-efficacy beliefs (Bandura, 1997). Self-efficacy beliefs describe the beliefs about one’s own capability to execute a given task despite possible difficulties (Tschannen-Moran & Woolfolk Hoy, 2001). Both teachers’ learning-related beliefs and self-efficacy beliefs are key aspects of their professional competences that serve as amplifiers and filters in bringing the professional knowledge of teachers into action (e.g. Gess-Newsome, 2015).

In the last decades, a lot of research has been carried out on effective PD. PD courses should take into account teachers’ needs (Guskey, 2002) and challenges that teachers face (Maass, 2011). They should also combine phases of learning in seminars with phases of learning at school (Lipowsky & Rzejak, 2015) and initiate cooperation between teachers (McLaughlin & Talbert, 2006). Naturally, they need to be relevant to teaching practice (Clarke, 1994) and should foster teachers’ reflection on their beliefs about mathematics and science teaching as well as on their teaching experiences (Tirosh & Graeber, 2003). These criteria for PD courses have proven effective in practice (see, e.g. Maass & Engeln, 2018, 2019) and provide guidelines for the design of PD courses on a general level.

### The Project MaSDiV and its Teaching and PD Concept

MaSDiV aimed to tackle the societal challenges mentioned in the introduction by developing research-based classroom and PD materials as well as delivering related PD courses suited to linking science and mathematics education with citizenship education. The PD course was designed to promote citizenship education and handling diversity in mathematics and science education by using IBL in contexts with SSI invoking modeling activities and intercultural learning. To this end, our materials included controversial questions such as “Do we improve the environmental situation of our planet by buying food in zero-waste-shops?”, “Should judges solely rely on DNA-tests in criminal cases in court?”, or “Do we fight social exploitation with buying fair-trade products?”

Answering such questions dealing with SSI involves not only mathematics, but also modeling real-life contexts, including balancing controversial aspects, and dealing with incomplete information (Ratcliffe & Grace, 2003). Additionally, students also need to form an opinion and make decisions about ill-defined problems (Zeidler & Nichols, 2009). Those decisions will, to some extent, depend on cultural and social peculiarities as well. For details about our classroom approach, we refer to Maass et al. (2019a).

The PD course is structured into three modules. In module 1, we introduce the concept of IBL and show its potential to deal with achievement-related diversity. Module 2 extends IBL to contexts in math and science education and discusses SSI. Module 3 embeds intercultural aspects in IBL. In this paper, we focus on module 2 since this module centers on the use of real-world problems and contexts in mathematics and science (for an overview, see Maass et al., 2019a).

The aims of module 2 can be summarized as follows:

- Understand the value of using a modeling, SSI, and IBL approach in science and mathematics and apply this in classroom teaching.
- Understand how modeling, SSI, and IBL in science and mathematics can support active citizenship.
- Understand the nature, applications, and implications of science and mathematics, including moral and ethical issues, for societies.

To reach these goals, the MaSDiV model for PD draws on principles for quality PD (see 2.4) and explicitly deals with the challenges teachers might have to face (such as time issues, curriculum, assessment, and classroom management) when implementing our concept of science and mathematics teaching.

Considering the time needed for an effective change of day-to-day teaching and taking into account the importance of both learning in the seminar as well as learning at school, we offered long-term PD courses. Several PD days were spread over a longer period of time. Our PD activities involved cycles of analysis, implementation, and reflection. *In the analysis phase* (during the PD course), teachers worked on collaborative classroom activities that made them familiar with the challenges of connecting science and mathematics education with citizenship education. Methods used in the module allowed teachers to work in groups, to investigate problems, to reflect on issues, and to discuss open questions. In other words, teachers participating in the course experienced the IBL approach themselves, whilst working on contexts relevant to society.

*In the implementation phase at school*, teachers were encouraged to adapt and use the activities in their own classrooms. We encouraged them to visit and observe each other’s lessons in order to give further opportunities for feedback and reflection. Finally, *in the reflection phase* (back in the PD course), teachers shared their classroom experiences (including both positive experiences as well as challenges), discussed the pedagogical implications, and reflected on the growth of new teaching practices and beliefs. This cyclic process was repeated at each PD event, as new pedagogical issues were addressed.

Module 2 consisted of five main activities. In activity 1, teachers reflected on their experiences and reasons for using contexts and modeling tasks to start from teachers’ competences and needs.

In activity 2, teachers worked on the topic “Can the earth feed us?”, a topic of high relevance for our societies in which the controversy between eating preferences and availability in rich countries on the one hand and feeding all humans across the world on the other hand is studied. Consequently, this task can be used by teachers in their day-to-day teaching based on subject-specific content that can be related to this context.

In activity 3, teachers analyzed further similar tasks, discussing a range of different possibilities to include SSIs into their own teaching to facilitate implementation. In activity 4, participants were asked to design a lesson using an SSI (to connect to practice and combine phases of learning in seminar and school) and reflect (activity 5) on drawbacks of the use of contexts and how to overcome the drawbacks.

All six partner teams (from Cyprus, Germany, Malta, the Netherlands, Spain, and Turkey) used the international materials and adhered to the overall pedagogical principles. Naturally, with six countries involved, we also allowed partners to adapt the course to local needs, but ensured that this PD course included at least 14 in-person hours for the participating teachers. In this respect, the PD course of the Spanish team represents a special case for the implementation of the PD course. Since Spain is a rather large country and participation was open for all science and mathematics teachers in Spain, a summer course was implemented. To also ensure that the presented course could be implemented in practice and reflected upon, it was also accompanied by an online follow-up during several months. In this online phase, special emphasis was placed on transferring the pedagogical approach to teachers’ practice, fostering teachers’ exchange of ideas and experiences while building a professional learning community. Spanish teachers were asked to design their own classrooms activities based on controversial SSI taking place in their students’ life and to share their lesson plans and reflections with the other participating teachers. Furthermore, teachers were specifically asked to evaluate their colleagues’ lesson designs and to provide constructive feedback. These kinds of interactions were expected to sustain change and to strengthen teachers’ self-efficacy beliefs (Wolters & Daugherty, 2007). In this sense, the Spanish PD course represents a special case of the PD program and deserves special attention in its evaluation.

## Design of the Study

### Implementation of the PD Courses and Data Collection

The MaSDiV PD courses were implemented in all six partner countries. In order to take the different national contexts into account, we first carried out a desktop analysis of the educational contexts in each country in which MaSDiV was to be implemented. This analysis included analyzing curricula, examining methods used to assess student performance, and looking at the existing conditions for teachers to participate in PD. Based on the analysis of the context, national teams (consisting of members from universities, schools, school authorities, and teacher education institutes) developed concepts for the adaptation of the international model. Subsequently, the national implementations varied to some extent (mainly in regard to the organizational framework), whilst supporting the major topics and major pedagogical principles as described above.

We ensured quality and consistency across countries with the following steps: (1) all courses were based on the international MaSDiV PD course materials; (2) we discussed the overall PD principles and their implementation at the biannual international project meetings; and (3) the PD course leaders were all either members of the international consortium or colleagues of involved consortium members and as such informed by them.

In 2018 and 2019, 453 teachers took part in the PD courses, all of them having applied for participation in the course. Across all countries, the course was based on the same materials and applied the same pedagogical principles. For the course, 14-h learning off-job were foreseen in each country. As we had to take into account the national contexts, the actual timeframe varied across countries for organizational reasons. In each of the six MaSDiV countries, a pre-post study was conducted to gain insight into the development of participating teachers during the MaSDiV intervention. Teachers filled out a questionnaire at the beginning of the first PD course session and at the end of the last session. For the Spanish teachers, the post-test was administered at the end of the online follow-up, which again emphasizes that the Spanish course deserves special attention.

### Design of the Evaluation Questionnaire

In order to assess the development of participating teachers during the MaSDiV PD, a quantitative pre-post-design was used. The questionnaires included descriptive background variables, items on the perception of the professional development program as well as sections about teachers’ self-efficacy beliefs, teachers’ learning-related beliefs, and teaching practices. The background variables comprised demographic information such as age, gender, subject, and previous formal learning experience of IBL. Items were assessed on a four-point Likert-scale whenever appropriate.

#### Perception of the MaSDiV PD.

In the post-questionnaire, a total of 15 items were designed to determine how participants experienced the MaSDiV PD. Following the theoretical discussion on effective PD, the items on the perception of PD were carefully chosen to cover key quality criteria such as the satisfaction with the experience, the utility and application of the content (adapted based on Grohmann and Kauffeld, 2013), collaboration during professional development (based on Cordingley et al., 2005), as well as the role of materials as a resource (Donna & Hick, 2017; Grossman & Thompson, 2008).

An exploratory factor analysis was conducted to determine underlying factors in the answers from the participants. Since the number of possible factors in exploratory factor analyses is not predetermined, we relied on Kaiser’s criterion (see also Field et al., 2012; Kaiser, 1960) to determine the number of factors. According to Kaiser’s criterion, only those factors that represent a substantial amount of variance should be retained, which is characterized by an eigenvalue greater than 1. Based on this procedure, a two-factor structure was identified. Since we expected that different aspects of the perception of the PD would be correlated, we used promax rotation for our principal factor solution to improve the interpretation of factor loadings (Field et al., 2012). Five items showed substantial loadings (> 0.30) on both factors and were therefore excluded from the following analysis. The remaining items were used to characterize the two factors. Based on the items with the highest loading, the identified factors can be described as representing (1) an overall satisfaction with the PD and (2) the provision of applicable materials. The factor loading matrix for the two-factor solution is presented in Table 1. Both scales also showed good to very good reliabilities (Cronbach’s αsat = .89 and Cronbach’s αmat = .73).

**Table 1:** Overview of the scales for the perception of the professional development

Item	Satisfaction	Applicable Materials
I enjoyed the PD	**0.58**	0.25
I got new input for my teaching	**0.78**	− 0.04
I learned a lot of new things in the PD	**0.86**	− 0.08
I successfully manage to apply the PD contents in my everyday teaching	0.02	**0.75**
I would recommend this PD to my colleagues	**0.71**	0.12
The PD supported collaboration and exchange with other teachers	**0.54**	0.05
The collegial professional exchange during the CPD promoted my individual learning	**0.63**	0.02
This PD experience will be useful in my work	**0.73**	0.09
During the CPD I was provided with teaching material.	0.11	**0.41**
I applied the teaching materials from the CPD in my everyday teaching	− 0.08	**0.93**

*Note.* The highest factor loading for each item is highlighted in bold

#### Teachers’ Self-Efficacy Beliefs, Learning-Related Beliefs, and Practices.

As outlined in the theoretical background, teachers’ self-efficacy beliefs, learning-related beliefs, and practices are key outcome measures of effective PD and drivers of sustainable change. Thus, teachers’ self-efficacy beliefs, learning-related beliefs, and practices were assessed with a paper-pencil test in the pre- and post-test (for details, see Sorge, 2020). To ensure the feasibility of the study during a PD course, all developed scales needed to be rather parsimonious whilst also allowing a reliable assessment. All items can be found in the “Supplementary Information.”

Teachers’ self-efficacy beliefs regarding the use of contexts relevant to society were assessed with four items (sample item: “I feel confident that I can facilitate classroom discussions about issues relevant to society.”). The construction of the items focused on assessing the confidence in one’s own capability to implement contexts in classrooms successfully (e.g. Bandura, 1997; Pajares, 1996). Teachers’ learning-related beliefs were also assessed with a scale consisting of four items (sample item: “Using contexts relevant to society allows students to develop a deeper understanding.”). The scale was also newly developed and focused on teachers’ explicit notions about effective teaching and learning (e.g. Kleickmann et al., 2016) with a specific focus on the use of contexts relevant to society. Teaching practices regarding the use of contexts relevant to society were assessed with a newly developed self-report instrument of three items (sample item: “In my teaching my students discuss controversial issues relevant to society.”). All scales show good reliabilities (α_LRB_ = .80, α_SEB_ = .79, α_TP_ = .74).

### Sample

From the total of 453 teachers who participated in the PD, matching pre-post data sets were available for *N* = 311 mathematics and science teachers from the six European countries Malta, Turkey, the Netherlands, Spain, Cyprus, and Germany. Sixty-six percent of the teachers were female and overall had a mean age of 39 years (SD = 11 years). The average teaching experience was 17 years (SD = 12 years). The overview of the sample is shown in Table 2. Overall, 49% of the participants were mathematics teachers and 51% taught science at school.

**Table 2:** Overview of the sample and subject distribution

	CY	GE	MT	NL	SP	TR
N	35	35	81	57	53	50
Mathematics	38%	100%	51%	31%	29%	56%
Science	62%	0%	49%	69%	71%	44%

### Analysis Strategies

To analyze to which degree teachers’ self-efficacy beliefs, learning-related beliefs, and practices changed across the MaSDiV PD and which factors contributed to that change, we used paired *t*-tests as well as multiple regression analyses. A paired *t*-test allows the comparison of results from the pre-test data with teachers’ answers from the post-test and indicates if teachers answered both tests significantly different. To determine the influencing factors, a multiple regression analysis was used. A multiple regression analysis is a statistical method that estimates to which degree a variable is predicted by a combination of multiple predictors. In this way, a regression coefficient indicates the strength of its influence when all other predictors are held constant at the same time. Three different multiple regression analyses were specified with teachers’ self-efficacy beliefs, learning-related beliefs, and practices as dependent variables, and characteristics from the teachers and the PD as independent variables. All specifications and computations were carried out with the software R (The R Core Team, 2020).

## Results

The MaSDiV PD program was designed to support mathematics and science teachers to bring together their own mathematics and science teaching with civic education through (the use of) modeling socially relevant contexts. We investigated (i) how teachers experienced the professional development program, (ii) how the professional development program impacted core aspects of teachers’ professional competence and practice, and (iii) what factors contributed to the impact of the professional development program.

### Perception of the Professional Development Program

**Fig. 2:**
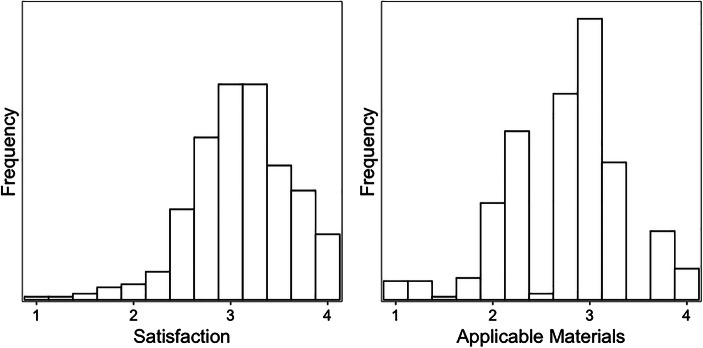
The perception of the PD course from participating teachers addressing **a** general satisfaction and **b** the provision of applicable materials

The *N* = 341 teachers that answered the post-test questionnaire showed a high overall satisfaction with their experiences during the professional development program, with a mean value of 3.11 (*SD* = 0.52). Furthermore, as indicated in Fig. 2a, 90% of the teachers rated their experiences above a value of 2.5, indicating that they are either satisfied or even highly satisfied. Teachers also rated the quality and amount of applicable materials with a mean value of 2.75 (*SD* = 0.60). Figure 2b shows that 69% of the participating teachers agreed or strongly agreed with the quality and amount of applicable materials. Accordingly, 31% of the teachers rate the quality and amount of applicable materials below 2.5 (i.e. as not sufficient enough).

### Changes in Teachers’ Self-Efficacy Beliefs, Learning-Related Beliefs, and Practices

To investigate changes in teachers’ self-efficacy beliefs, learning-related beliefs, and practices after the completion of the professional development program, we used data from *N* = 311 teachers from the pre- and post-test. Table 3 summarizes the findings from the comparison between pre- and post-test. The comparison shows that participating teachers’ self-efficacy beliefs, learning-related beliefs, and practices were changed significantly. Teachers reported significantly higher self-efficacy beliefs after the professional development (*M* = 2.99, *SD* = 0.43) compared to the beginning of the professional development program (*M* = 2.80, *SD* = 0.48), with a medium effect size of Cohen’s *d* = .41, *t*(303) = 7.19, *p* < .001. Teachers’ learning-related beliefs increased significantly with a small effect size of Cohen’s *d* = .18 from the first meeting (*M* = 3.25, *SD* = 0.42) to the last meeting (*M* = 3.32, *SD* = 0.45), *t*(303) = 3.13, *p* < .01. It must be noted that teachers already had rather strong beliefs about the benefits of using contexts for students’ learning in the first meeting of the PD course with a mean value of 3.25.

**Table 3:** Teachers’ self-efficacy beliefs, learning-related beliefs, and teaching practice from the pre- and post-test including t-value from paired t-test and Cohen’s d

**Scale**	**Pre**	**Post**	***t***	***d***
Self-efficacy beliefs	2.80 (0.48)	2.99 (0.43)	7.19***	0.41
Learning-related beliefs	3.25 (0.42)	3.32 (0.45)	3.13**	0.18
Teaching practice	2.18 (0.63)	2.38 (0.66)	5.63***	0.32

*Note.* *** *p* < .001, ** *p* < .01

Finally, the participating teachers also indicated that they use socially relevant contexts significantly more frequently after completing the MaSDiV professional development program (*M* = 2.38, *SD* = 0.66) compared to their practice at the beginning of the program (*M* = 2.18, *SD* = 0.63), *t*(301) = 5.63, *p* < .001. The change in teaching practice had an effect size of Cohen’s *d* = .32.

### Influencing Factors for the Development of Teachers’ Self-Efficacy Beliefs, Learning-Related Beliefs, and Practices

In a third step, we investigated which influencing factors regarding the teachers, their school, and the professional development, contributed to the outcomes of the professional development program. In Table 4, influencing factors in regard to teachers and the professional development program are separated by a vertical line.

**Table 4:** Multiple regression model investigating the impact of teacher and PD characteristics on teachers’ self-efficacy beliefs, learning-related beliefs, and practices

	Self-efficacy Beliefs	Learning-related beliefs	Practices
Prior beliefs/practices	0.45***	0.38***	0.48***
Prior IBL learning	0.07	−0.11*	0.01
Cultural diversity at school	− 0.11*	−0.14**	− 0.01
Spain summer school	0.03	0.11*	0.12*
Satisfaction with PD	0.14*	0.05	0.06
Applicable materials	0.06	0.11	0.17**
*R*^2^	0.25	0.24	0.32

*Note.* *** *p* < .001, ** *p* < .01, * *p* < .05

First, *characteristics of the teachers* influenced the results. Our results show that for teachers’ self-efficacy beliefs, learning-related beliefs, and practices the respective pre-test results have a significant influence on teachers’ post-test scores (β_SE_ = .45, β_BE_ = .38, β_PR_ = .48, *p* < .001 respectively). Additionally, teachers with no prior learning experiences about IBL report stronger learning-related beliefs at the end of the program (β = −.11, *p* = .03).

Second, *characteristics in relation to the school* influenced the results. Teachers who rate the cultural diversity at their own school as high have weaker self-efficacy beliefs and learning-related beliefs after completing the program (β_SE_ = −.11, *p* = .04, β_BE_ = −.14, *p* < .01).

Third, *characteristics related to the professional development program* itself also impact the outcome of the professional development program significantly. For example, Spanish teachers attending the PD with continuous online support (as described in part 3) reported stronger learning-related beliefs and use contexts more frequently in their own classroom compared to teachers who followed a shorter professional development approach (β_BE_ = .11, *p* = .03, β_PR_ = .12, *p* = .01). Additionally, teachers who were more satisfied with the quality and collaborations during the professional development course also showed higher self-efficacy beliefs to actually enact the content taught in their own classroom (β = .14, *p* = .03). Finally, the rating of the amount and quality of applicable materials for classroom use also influenced the frequency of the use of contexts positively (β = .17, *p* < .01).

Through the combination of teacher, school, and PD characteristics, we were able to explain a substantial amount of 32% of the variance for teachers’ post-test answers on their practices. For teachers’ self-efficacy beliefs and learning-related beliefs, the regression model explains 25% and 24% of the variance respectively.

## Discussion

One of the main objectives of schools is to prepare students to become active democratic citizens for our society (European Commission/EACEA/Eurydice, 2016). Key aspects of an education that supports the development of active citizens are to provide students with the necessary knowledge to make informed decisions, show the relevance of such knowledge for meaningful problems, and involve students in the practices of generating such knowledge. More specifically, modeling activities as well as addressing socio-scientific issues using a student-centered approach such as IBL can be identified as the necessary tools to combine science and mathematics with citizenship education. However, neither of these three tools are frequently applied in today’s classrooms (Maass et al., 2019b). To support teachers in incorporating citizenship education in science and mathematics classrooms more frequently, there is a strong need for additional PD courses (Knippels & van Dam, 2017). We therefore developed a new PD course as part of the MaSDiV project and investigated to which degree key characteristics of teachers changed after participating in that course.

In general, 90% of all teachers rated that they were satisfied or even highly satisfied with the PD course and their own growth. The rating of general satisfaction with the PD course also correlated with the degree teachers’ self-efficacy increased during the PD. Teachers who felt that they learnt something during the PD and had valuable opportunities to discuss their ideas with other teachers also reported significant higher self-efficacy beliefs after the PD course.

The feedback on the quality and availability of applicable materials as one quality indicator of the PD was more mixed compared to the feedback on general satisfaction. While a majority of 69% of the teachers also agreed or strongly agreed that they received high-quality materials, a substantial number of teachers were less satisfied with the provided materials. This feedback from the teachers suggests that there is a need to increase the number of ready-to-use materials in the course. This is in line with other research that highlights that teaching and curricular materials affect instructional practice and student learning (Ross et al., 2003; Schoen et al., 2003). The importance of applicable materials, as emphasized by teachers, is also reflected in its impact on teachers’ practices. Our regression analysis shows that teachers who felt that they received high-quality applicable teaching materials also used contexts in their own classroom more frequently. An increase of applicable materials for the PD course could therefore have an impact on teachers’ actual practice in their own classrooms.

Our results show that teachers’ self-efficacy beliefs about using relevant contexts, their learning-related beliefs about the benefits of using relevant contexts, as well as their own teaching practice changed significantly after participating in the MaSDiV PD course. The strongest impact of the PD course was found for teachers’ self-efficacy beliefs. In general, after participating in the MaSDiV program, teachers felt better prepared to implement the desired way of teaching The change in teachers’ learning-related beliefs was significant too, although with a small effect size. Previous research already echoed that teachers’ learning-related beliefs are rather hard to change since they are stable over time (Törner, 2002). In addition to that, the participating teachers already had rather strong beliefs about the benefits of using contexts for student learning and, thus, less room for improvement. Since teachers applied to participate in the PD course, it was not surprising that they already had positive attitudes towards the teaching approach presented in the PD. Our results should therefore not be generalized to the general population of science and mathematics teachers. Based on our initial results, future research should extend the investigation to learning-related beliefs about the benefits of using contexts to science and mathematics teachers in general.

Additionally, our results also indicate that the course in Spain had an additional impact on teachers’ learning-related beliefs and practices. A possible explanation could be that Spain provided substantially longer online support of the PD program, with a special emphasis on transfer to teaching practices as well as promoting the exchange of ideas and experiences among teachers in a professional learning community.

Most importantly, our pre-post-test comparison showed that participating teachers indeed reported that they use socially relevant contexts more frequently compared to the beginning of the PD course. Consequently, a well-planned PD can support teachers in applying even complex teaching approaches such as citizenship education in mathematics and science.

Our results further show that the MaSDiV PD impacted teachers’ self-efficacy beliefs, learning-related beliefs, and practices differently based on their personal characteristics, their school characteristics, and the specifics of the PD course itself. *First*, we controlled for teachers’ entry characteristics in the regression analyses. The change in teachers’ learning-related beliefs differed based upon teachers’ prior learning opportunities. Teachers with no learning opportunities on IBL prior to the PD course had significantly stronger learning-related beliefs at the end of the course. Thus, the MaSDiV PD course had a substantial impact especially for teachers with limited prior knowledge on the topics at hand.

*Second,* another factor contributing to the impact of the PD course was the perceived cultural diversity at the teachers’ own school. Teachers who reported a high cultural diversity in their own school had significant weaker self-efficacy beliefs and learning-related beliefs compared to teachers with less cultural diversity. Differently perceived cultural diversity can serve as diverse focal points of teachers’ development during the PD course (see, e.g. Buehl & Beck, 2015). Module 3 of the MaSDiV PD course focused explicitly on addressing cultural diversity in science and mathematics classrooms. Thus, it could be that teachers who reported high cultural diversity in their own school focused less on the use of contexts for citizenship education. Another explanation could be that teachers perceiving high cultural diversity in their school intend to focus on basics in mathematics and science education, since contexts often require complex speech comprehension, which is known to be one of the main error sources (Radatz, 1979, p. 168). In addition, cultural characteristics may also impact the reception of contexts.

*Third,* we attend to the characteristics of the PD course. Our results underline the need for long-term PD courses. Teachers who attended an extended summer school in Spain had significantly stronger learning-related beliefs about the benefits of using contexts and reported a more frequent use of contexts in their own classrooms. Compared to the relatively shorter PD version in other countries (which still comprised several days across multiple months), the summer school in Spain allowed the participants to discuss the content of the PD for a full week and provided them with an extended opportunity to try out the practices after the summer course, with the possibility to discuss their experiences with other teachers and PD leaders online. Seemingly, this extended support and ‘try-out’ phase enabled the teachers to change their teaching practice consistently and also reflect on those experiences, which caused an additional change in teachers’ learning-related beliefs (Guskey, 2002). Therefore, one measure to enlarge the impact of a PD course on teachers might be to provide teachers with extended support and opportunities for “trying out” in order to change their teaching practice towards citizenship education.

While the implication of long-term PD courses is supported by the data, some limitations need to be taken into account when interpreting its results. As mentioned before, teachers participated voluntarily in the PD courses in all countries. Thus, our results have to be interpreted with caution since voluntarily participating teachers are expected to be more motivated and have a strong motivation to work on their own teaching practice. However, the system of professional development across many countries in the EU and worldwide depends on voluntary participation and engagement of teachers. Policy makers should therefore support and incentivize PD participation across all countries so that in-service teachers have the possibility to work on their own teaching practice. Additional research is also necessary in regard to what types of incentives for PD participation teachers need to enroll in programs such as MaSDiV.

A second limitation is the paper-pencil test employed in this study. Such a paper-pencil test relies heavily on the perception of teachers’ own practice as well as their reflection on their own beliefs. While such a paper-pencil test certainly provides valuable insights and is an ecological assessment of teachers’ beliefs and practices, additional research is necessary. This could involve using students’ assessments of teaching practices as well as qualitative data to gather a more complex picture of teachers’ belief systems (Luft & Roehrig, 2007). This has been done, for example, within the project Primas, in which a PD course on inquiry-based learning was developed and implemented. Here, students’ data supported the perspective of teachers (Maass & Engeln, 2018).

Despite those limitations, our study provides a sound framework in regard to how science and mathematics instruction can be used to also support citizenship education. Furthermore, the framework consisting of inquiry-based learning, modeling activities, and SSI was used to design and implement a PD course in multiple countries in Europe. The results of our research show that the developed PD concept was successful in supporting the development of teachers’ self-efficacy beliefs, learning-related beliefs, and practices. Future PD courses that try to address topics such as the use of contexts should focus on providing selected high-quality and ready-to-use materials in order to ensure a change in teaching practice. A prolonged support of teachers who participated in a PD through an online environment has also proven to be a significant contributor for teachers’ development. With such long-lasting support through PD and additional incentives from policy makers, teachers can be empowered to design lessons that not only focus on knowledge transmission, but also on helping students to become active members in our societies.

## Supplementary Information

ESM 1 (DOCX 14 kb)
