# Supplementary material for: Promoting Active Citizenship in Mathematics and Science Teaching
Source: Int J Sci Math Educ. 2021 Jun 19;20(4):727–46. doi: 10.1007/s10763-021-10182-1 (PMC8214369; doi:10.1007/s10763-021-10182-1)
Supplement: Supplementary file 1 — (DOCX 14 kb) [file 10763_2021_10182_MOESM1_ESM.docx]

Appendix

**Learning-related beliefs**

To what extend do you agree with the following statements?

|  | Strongly disagree | Disagree | Agree | Strongly agree |
| --- | --- | --- | --- | --- |
| Science and math instruction needs to address issues relevant to society to foster students’ values. | 🖵 | 🖵 | 🖵 | 🖵 |
| Using contexts relevant to society allows students to develop deeper understanding. | 🖵 | 🖵 | 🖵 | 🖵 |
| Instruction needs to provide students with opportunities to discuss issues relevant to society. | 🖵 | 🖵 | 🖵 | 🖵 |
| Situating activities in contexts relevant to society will help improve students’ ability to make justified decisions. | 🖵 | 🖵 | 🖵 | 🖵 |

**Self-efficacy beliefs**

To what extend do you agree with the following statements?

| I feel confident that … | Strongly disagree | Disagree | Agree | Strongly agree |
| --- | --- | --- | --- | --- |
| …I can incorporate issues relevant to society in my teaching. | 🖵 | 🖵 | 🖵 | 🖵 |
| …I can develop teaching activities addressing issues relevant to society. | 🖵 | 🖵 | 🖵 | 🖵 |
| …I can use inquiry-based activities to engage students in dealing with issues relevant to society. | 🖵 | 🖵 | 🖵 | 🖵 |
| …I can facilitate classroom discussions about issues relevant to society. | 🖵 | 🖵 | 🖵 | 🖵 |

**Inquiry-based teaching practice**

How often do the following activities take place in your lessons?

| In my teaching … | Never or hardly ever | Some lessons | Most lessons | Almost every lesson |
| --- | --- | --- | --- | --- |
| …my students discuss controversial issues relevant to society. | 🖵 | 🖵 | 🖵 | 🖵 |
| …I use contexts to promote fundamental values (e.g. democratic values). | 🖵 | 🖵 | 🖵 | 🖵 |
| …I use contexts relevant to society. | 🖵 | 🖵 | 🖵 | 🖵 |
